# Alkyl Gallates as Potential Antibiofilm Agents: A Review

**DOI:** 10.3390/molecules28041751

**Published:** 2023-02-12

**Authors:** Mahmoud Rayan, Saleh Abu Lafi, Mizied Falah, Tomas Kacergius, Agne Kirkliauskiene, Vika Gabe, Anwar Rayan

**Affiliations:** 1 Holy Family Hospital, Nazareth 16000, Israel; 2 Faculty of Pharmacy, Al-Quds University, Abu-Dies 144, Palestine; 3 Institute for Medical Research, Holy Family Hospital, Nazareth 16000, Israel; 4 Department of Physiology, Biochemistry, Microbiology and Laboratory Medicine, Institute of Biomedical Sciences, Faculty of Medicine, Vilnius University, 03101 Vilnius, Lithuania; 5 Faculty of Science, Al-Qasemi Academic College, P.O. Box 124, Baka EL-Garbiah 30100, Israel

**Keywords:** alkyl gallate, natural product, antimicrobial, biofilm

## Abstract

Biofilms, which consist of microorganisms embedded in a polymer-rich matrix, contribute to a variety of infections and increase antimicrobial resistance. Thus, there is a constant need to develop new chemotherapeutic agents to combat biofilms. This review article focuses on the use of alkyl gallates, gallic acid and its esters (methyl, ethyl, propyl, butyl, hexyl, octyl, and dodecyl gallate), most of which are found in plants, to inhibit biofilm formation. The studies under review reveal that alkyl gallates have the capacity to prevent biofilm development and eradicate mature biofilms through mechanisms that suppress the synthesis of the extracellular polymeric matrix, inhibit quorum-sensing signaling, and alter the microbial cell membrane. The effects are stronger the greater the length of the alkyl chain. Moreover, the alkyl gallates’ preventive activity against biofilm formation occurs at doses below the minimum inhibitory concentration. More importantly, combining alkyl gallates with antimicrobials or blue-light irradiation produces a synergistic effect on the inhibition of biofilm formation that can be used to treat infections and overcome microbial resistance.

## 1. Introduction

Biofilms are an aggregation of one or more types of microbial cells that grow on a wide variety of surfaces and play a significant role in the persistence of bacterial infections. Bacterial biofilms are involved in many infections that contribute to pathogenesis, thereby imposing heavy economic pressure on the healthcare sector [1,2].

**Figure 1:**
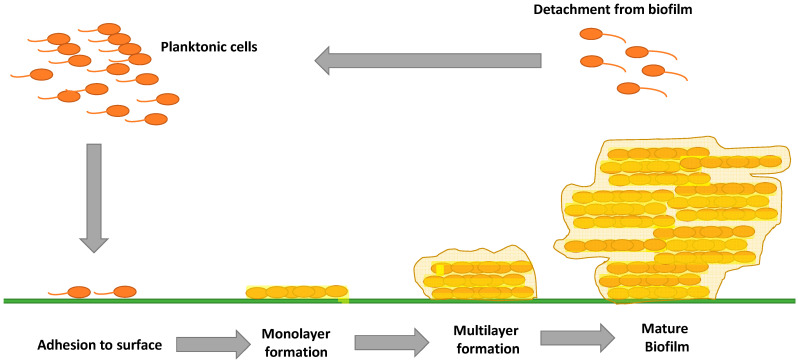
Sequence of stages of bacterial biofilm formation.

Biofilm formation involves a sequence of stages: At first, there is an initial reversible adhesion of planktonic (free-floating) bacteria to the surface in question, followed by the formation of a monolayer of film that produces an extracellular matrix (Figure 1) which consists of polysaccharides, proteins, lipids, and other biochemicals [3]. The biofilm grows in three dimensions, and the attachment becomes irreversible. The resulting bacterial resistance to antibiotics is several orders of magnitude greater than that of planktonic bacteria [4]. In the last stage, a portion of the mature cells start to detach and disperse into the environment as planktonic cells to potentially start a new cycle of biofilm formation [5].

Dental biofilms play an important role in tooth decay. The control of their formation, therefore, is a major goal of dental specialists. A common example of dental biofilm is dental plaque, which is a bacterial colony that forms on the surface of a tooth, mainly involving *Streptococcus mutans*, which has the ability to produce a biofilm and organic acids (acidogenicity) from dietary sucrose, causing tooth decay, which is one of the most prevalent oral diseases [6,7].

The resistance of bacteria in biofilms to antibiotics is 1000-fold greater than that of their planktonic counterparts [4]. This elevated bacterial resistance to antibiotics and conventional treatment has shifted the attention of the medical research community to natural sources capable of preventing oral bacterial growth, adhesion, and colonization [8]. Natural compounds, such as essential oils, can affect biofilm formation by inhibiting peptidoglycan synthesis, damaging microbial membrane structures, and modulating quorum sensing [9].

Natural products, including some found in medicinal plants, have been proven to contain important secondary metabolites that may be used to effectively inhibit the growth of pathogenic bacteria [10,11,12,13], particularly *Streptococcus* biofilm formation [8]. Phytochemicals from plants include antibacterial polyphenols, alkaloids, flavonoids, quinones, tannins, coumarins, terpenes, and saponins [1,8]. The anticolonization effects of natural compounds follow from the inhibition of bacterial attachment through alteration of the physicochemical properties of the cell surface [14].

**Figure 2:**
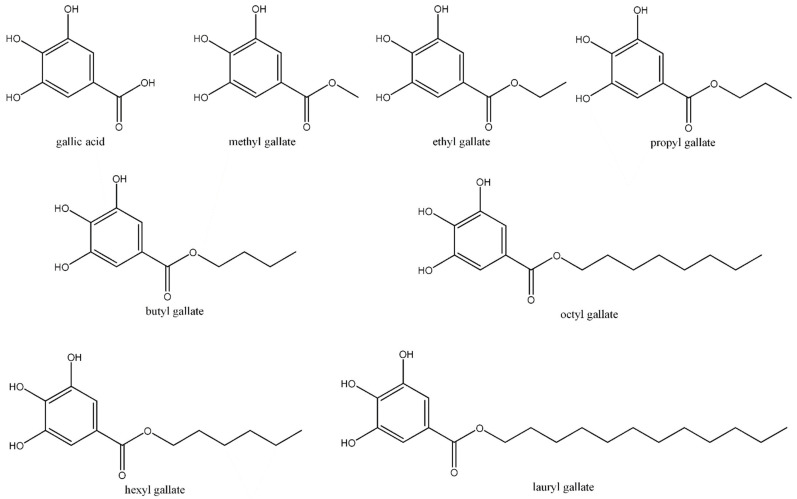
Chemical structure of alkyl gallate derivatives.

Polyphenolic gallates are secondary metabolites, most of which are derived from plants and are made up of esters of gallic acid. In this review article, we focus on reviewing the antibiofilm effect of alkyl gallates (see Figure 2). 

Polyphenolic gallic acid and epigallocatechin gallate, which are the most abundant catechins in green tea, have been investigated for their effects on dental diseases [15,16,17]. Both are natural compounds that inhibit the biofilm formation of *Streptococcus mutans* by inhibiting its attachment capability [17,18]. It is believed that gallic acid and epigallocatechin gallate influence the colonization of abiotic surfaces through a variety of mechanisms, namely by affecting the physico-chemical properties of the cell surface, inducing changes in the shape of cell envelopes, and causing a calcium efflux [18]. Moreover, natural polyphenol has a significant inhibitory effect on dental biofilm formation by *Streptococcus mutans* and thus might be a good candidate drug for preventing dental caries [17]. 

## 2. Gallic Acid

Gallic acid (GA), found in many plant extracts, is a natural phenolic compound with potent antioxidant and antimicrobial actions against various pathogenic bacteria [19,20,21,22]. Many studies have documented its antimicrobial effect on biofilm formation and its ability to combat pathogenic bacteria [19,20,21,22]. Biofilm formation in bacteria is closely associated with the threat of infection and is a major mechanism of resistance against antimicrobial agents. GA actions against biofilm formation and the dispersal of bacteria has been studied in respect of *Proteus spp.* , *Escherichia coli*, *Pseudomonas spp.* , *Salmonella spp.* , *Streptococcus mutans*, and *Staphylococcus aureus*, as well as other multispecies bacteria [19]. At a concentration of 100–200 mg/L, GA can reduce bacterial growth by 86%, biofilm formation by 85.5%, and extracellular bacterial polysaccharide by 88.6%, but has no effect on pre-formed biofilm. Silby et al. [23] found that GA can inhibit the biofilm formation of clinical isolates of *Pseudomonas aeruginosa*, a bacterium that colonizes diverse environmental niches with strong adaptability and modulates the host’s immune response, causing many nosocomial infections. The formation of biofilm and the associated synthesis of pyomelanin in these bacteria confer the ability to acquire a drug-resistant pathogenic phenotype [24,25]. GA was found to induce pyomelanin synthesis and inhibit biofilm formation in two isolates of *Pseudomonas aeruginosa* in a concentration-specific manner. The inhibition (0.4-fold) was observed at a 5-mg/mL concentration when GA was tested before the initiation of biofilm formation. Stronger inhibition (0.6–0.75-fold) was observed at a 3–5-mg/mL concentration when GA was tested on pre-existing biofilms. *P. aeruginosa*, which is resistant to ampicillin in both planktonic and biofilm forms, is sensitized by treatment with GA (3 mg/mL) due to membrane damage [26]. 

Moreover, GA has shown significant antimicrobial activity by inhibiting the adhesion and reducing the acidogenicity and aciduricity of biofilms of *S. mutans*, and by downregulating the expression of glycosyltransferase genes (*gtfB*, *gtfC*, and *gtfD* genes) in *S. mutans* biofilms [21]. Its minimum inhibitory concentration (MIC) is 250 µg/mL, and GA extracted from the *Libidibia ferrea* plant, which constitutes 29.45% of the phytoconstituents, has a minimum bactericidal concentration (MBC) of 1000 μg/mL [21].

The antibacterial effects of GA have also been investigated for synergy with other agents. As mentioned, GA by itself has shown strong activity against *S. mutans*, inhibiting its biofilm formation and markedly enhancing the anti-*S. mutans* activity of the polypeptide nisin, decreasing the value of its MIC eightfold in a bacteriostatic manner [27]. However, Wang and Lam [18] found that GA also enhanced the aggregation and biofilm formation of *Actinomyces naeslundii* on abiotic surfaces by affecting physicochemical properties of the cell surface and changing the shape of the cell envelopes, causing a calcium efflux from the bacteria. Furthermore, GA is a standard phenolic acid found in numerous foods; it inhibits toxicity by damaging the permeability of bacterial cells, which it accomplishes by changing the hydrophobicity of cell membranes and causing pore formation [28]; it displays antibacterial activity and has a MIC of 9.4 mM against strains of *Chromobacterium violaceum* [29]. Bali et al. [30] have shown that GA exhibits a MIC of 8.8 mM against *C. violaceum*; in their study that compared the inhibitory effect of GA on biofilm formation and quorum sensing (QS) (i.e., the microbial communication system) in *C. violaceum*, GA did not exhibit anti-QS activity; it inhibited only microbial growth in a sub-MIC range of 46.87–750 μg/mL, exhibiting its weakest biofilm inhibition from 1.38% ± 0.08% to 9.57% ± 0.06%.

The pathogen *Acinetobacter baumannii*, which shows extensive resistance to almost all antibiotics, is responsive to GA. Sherif et al. [31] showed that GA had antibacterial effects on *A. baumannii* at MICs ranging from 1.32 to 2.11 mg/mL and that subinhibitory concentrations (e.g., the ½ MIC) of GA induced 91% inhibition of biofilm formation; this study highlighted the association between enhanced biofilm formation and the resistance profile of *A. baumannii*. A biofilm of *A. baumannii* most likely acts as a barrier to antibiotic penetration, and GA, a phenolic acid, causes structural changes to the bacterial membrane that result in the leakage of essential intracellular constituents. Sowndarya et al. [28] tested crude extracts of GA, isolated from cashew nuts and coconut shells, on biofilms of *Ralstonia solanacearum,* a soil-borne plant pathogen, and found that a ½ MIC (200 μg/mL) of both extracts inhibited biofilm formation at means of 66% and 53%, respectively; however, a pure extract composed of GA compound that was isolated from cashew nut shells showed a MIC of 3 mg/mL against *R. solanacearum*. A sub-MIC concentration of 1.5 mg/mL inhibited 85% of the young biofilms and disrupted 86% of the mature ones, which was confirmed by crystal violet assay and electron microscopy [32]. In contrast, low activity of GA was recorded (MIC > 1 mg/mL) towards seven antibiotic-resistant strains of bacteria by Ivanov et al. [33]. GA, as a major component of extract fractions made from the bark of *Schinopsis brasiliensis Engl.*, has been found to have antimicrobial activity with a MIC of less than 1 mg/mL against oral microorganisms such *S. mutans*, *S. oralis*, *S. mitis*, and *S. salivarius* [34]. Although no direct tests were conducted on biofilm formation, these GA fractions could be candidates for dental formulations that inhibit biofilm formation. GA tested on *E. coli* pathotypes (e.g., enteropathogenic *E. coli* [EPEC], enterohemorrhagic *E. coli* [EHEC], and enterotoxigenic *E. coli* [ETEC]) [35] showed an inhibitory effect at concentrations of 2.1–2.2 mg/mL; at the minimum bactericidal concentration (MBC) and at sub-inhibitory concentrations (e.g., 1.5 mg/mL), GA reduced biofilm formation and the expression of biofilm-related genes. In a study by Kang et al. [36], the *E. coli* gene pgaABCD, which encodes the polymer poly-beta-1,6-N-acetyl-D-glucosamine (PGA), essential to biofilm formation by facilitating cell-to-cell adhesion and attachment to surfaces, was significantly inhibited by GA at a sub-MBC of 2 mg/mL. The MIC values determined for biofilm formation and biofilm eradication were 2 and 8 mg/mL, respectively, whereas the MIC and MBC values of GA against planktonic *E. coli* were found to be 0.5 and 4 mg/mL, respectively. The higher MIC for biofilm eradication strongly indicates that bacteria were embedded in a polymer matrix that increased the resistance of *E. coli* to GA. In a study by Teodoro et al. [37], in which it was used as a reference molecule, GA was able to inhibit the biofilm formation of *Candida albicans* at 2x the MIC (10 mg/mL) only after 48 h, with no effect on biofilm formation after 24 h.

GA, in combination with ampicillin, was shown to effectively inhibit the growth and biofilm viability of several multidrug-resistant bacterial clinical strains of *E. coli*. At a MIC of 1024 µg/mL, it enhanced the antibacterial activity of ampicillin, which strongly indicates that these antibacterial agents possess synergistic effects [38]. The inhibitory effect of GA, in combination with ampicillin, on the viability of *E. coli* biofilm, seen in confocal laser scanning micrographs, clearly demonstrates that the percentages of the biomass of dead biofilm after treatment with gallic acid-ampicillin (68.46%) were higher than the percentages of dead biofilm biomasses after treatment with the individual antibacterial agents (~42.4%). In a study by Gobin et al. [39], a combination of GA and carvacrol was found to work synergistically to increase the eradication of biofilm of *Pseudomonas aeruginosa* and to cause the complete eradication of *S. aureus* biofilm. The combined effects were strong and synergistic against both single- and dual-species mature biofilm formed by these strains of bacteria, which commonly cause infection in wounds and delay wound healing. GA and carvacrol completely inhibited the growth of these bacteria at MICs of 2.5 mg/mL and 0.128 mg/mL, respectively.

Overall, GA has been used successfully to slow and inhibit biofilm formation in various bacterial species, both alone and in combination with other agents. The results summarized in Table 1 strongly indicate that the inhibition of biofilm formation by gallic acid may serve as a novel strategy for targeting harmful bacteria and prove useful in food preservation and the pharmaceutical industry.

## 3. Methyl Gallate

Kacergius et al. [7] used an optical profilometry assay to evaluate the effects of sumac extract, specifically its bioactive component Methyl Gallate (MG), on biofilm formation by *S. mutans*. MG is the most active antibacterial compound in *Rhus coriaria* L. (sumac). It was found to reduce biofilm biomass on a solid polystyrene surface by 68–90%, to reduce the roughness and thickness of biofilm on glass by 99% (at 1 mg/mL), and to suppress acidogenicity. The effects were dose-dependent.

Hossain et al. [40] were the first to report the anti-quorum-sensing (QS) action of Methyl Gallate (MG) in *P. aeruginosa*. Of the five phenolic compounds investigated in the preliminary study for their interference with AHL-mediated QS, MG was the only one to exhibit a strong anti-QS effect, and was later found to inhibit QS in *Chromobacterium violaceum* by interfering with the synthesis and activity of AHL. In *Pseudomonas aeruginosa*, it suppressed the expression of genes that encode the virulence factors elastase, protease, exopolysaccharide, and rhamnose, and by interfering with swarming motility, prevented biofilm formation. It also affected QS in *P. aeruginosa* by significantly down-regulating, in a concentration-dependent manner, the expression of five QS-regulatory genes. MG had no significant toxic effects on the RAW 264.7 macrophage cell line at a concentration < 6.25 mg/mL.

Researchers have recently worked to incorporate antibacterial agents into dental adhesives. Yu et al. [41] experimented with introducing epigallocatechin-3-gallate (EGCG) and epigallocatechin-3-O-(3-O-methyl)-gallate (EGCG-3Me) separately into the commercial adhesive Single Bond 2 (SB 2), at concentrations of 200, 400, and 600 µg/mL, to test for antibacterial properties and bonding durability with dentin. The cured modified adhesives inhibited the growth of *S. mutans*, a concentration-related effect, and inhibited the bacterium’s adhesion to the dentin–resin interface, while bonding stability was maintained. The microtensile bond strength of the modified adhesives was stronger than that of SB 2 after 5000 cycles of thermocycling. The degree of conversion (DC) of the adhesive system varied with concentration and the depth of the hybrid layer. However, the EGCG-3Me preparation more strongly inhibited biofilm development, and the 400-µg/mL sample had antibacterial capabilities and boosted bonding stability without affecting the DC. On several points, including its antibacterial effect and its weaker influence on DC, the EGCG-3Me-modified adhesive performed better than the EGCG-modified adhesive.

Mechesso et al. [42] studied the application of MG in combination (MT) with the veterinary antibiotic Tylosin (Ty) to *Salmonella enterica Serovar* Typhimurium, a pathogen that causes gastrointestinal disease in both humans and animals. They assessed the effects of sub-inhibitory concentrations of MT on the bacterium’s viability, membrane potential, and cell integrity, its interactions with the host cell (adhesion, invasion, intracellular survival), and biofilm formation. The MT combination produced significant cell membrane damage, reduced membrane potential, and leakage of bacterial cell contents. Biofilm formation and the adhesive and invasive capabilities of *S.* Typhimurium were significantly impaired. Treatment of infected cells with MT up-regulated the gene expression of interleukin IL-6, IL-8, and IL-10 cytokines, which play crucial roles in defending host cells against *S.* Typhimurium. In sum, the MT combination of MG and Ty acted synergistically against the pathogen.

Bag et al. [43] studied the effects of MG isolated from *Terminalia chebula* (chebulic myrobalan) against the multidrug-resistant pathogen *Vibrio cholerae*, the cause of cholera. Inhibition of fluid accumulation and anti-colonization were examined in vivo in BALB/c mice; the intestinal inflammatory reaction induced by the pathogen was studied in Swiss albino mice, and biofilm inhibition was examined in vitro. MG was found to inhibit the biofilm formation of two isolates of *V. cholerae* (SG24 and PC4), with 70% inhibition at a MIC of 64 µg/mL. TEM analysis revealed that MG caused total disintegration of the inner and outer membranes of the pathogen and leakage of cytoplasmic material. Oral doses of 50 and 500 mg/kg body weight in two groups of experimental mice significantly inhibited pathogen-induced inflammation. Inhibition by MG was also observed against fluid accumulation and colonization.

In a search for substances with anti-pathogenic and anti-biofilm properties, Campbell et al. [44] assayed 13 phenolic compounds and identified 4-ethoxybenzoic-acid (4EB) and MG as targets of interest. Both inhibited biofilm formation up to as much as 87%, with minimal effect on the viability of stationary-phase cells or bacterial growth. While 4EB was found to synergistically potentiate the action of the antibiotic vancomycin against biofilm-dwelling cells, MG was not. However, MG exhibited anti-pathogenic and anti-biofilm activity and attenuated the growth of *S. aureus*.

Dávila-Aviña et al. [35] evaluated the effects of five phenolic compounds (PCs), including MG, on several characteristics of three *E. coli* pathotypes (enteropathogenic, enterohemorrhagic, and enterotoxigenic). They looked at effects on growth, swarming motility, biofilm formation, and the expression of selected virulence genes. MG, along with tannic acid (TA) and GA, showed bactericidal activity against all three pathotypes of *E. coli*; at low concentrations, MG had the strongest effect on all the strains. Additionally, at low concentrations, the three compounds affected virulence factors such as swarming motility and biofilm formation without significantly reducing cell populations. The authors concluded that the PCs under study had potential for controlling the growth, swarming motility, and virulence gene expression of all three pathotypes, but care should be taken to apply the proper concentrations to avoid inducing virulence factor genes. 

The results of the inhibition of biofilm formation by MG are summarized in Table 2.

## 4. Ethyl Gallate

Only a few papers have been published on the effects of ethyl gallate (EG) on biofilm formation. In early 2019, Gabe et al. [6] reported on EG as an antimicrobial agent that affected *Streptococcus mutans* with a MIC of 1.56 mg/mL (7.87 mM) and a MBC of 6.25 mg/mL (31.54 mM). It displayed antibiofilm activity and a dose-dependent inhibition of biofilm formation on polystyrene and glass surfaces, which was significant at all the concentrations tested; EG at a concentration of 3.53 mM inhibited biofilm formation on polystyrene by 68% and on glass by 91%, compared to untreated samples, and almost completely inhibited the acidogenicity of the biofilm, inducing a 95% decrease in pH levels. At a 0.39 mg/mL concentration (comparable to 25% of the MIC value), EG produced significant changes in the expression of the genes *gtfC* (a 98.6% increase in fold change), *gtfB* (a 47.5% increase), and *gbpB* (a 13.8% increase), but showed no significant expression changes for *gtfD*, *atpD*, and *atpF* compared to the control. 

Kim et al. [45] tested six alkyl gallate compounds for their effects on virulence factors and biofilm formation in *Pseudomonas aeruginosa*. EG was among the compounds that were effective at inhibiting virulence factor production and biofilm formation in *P. aeruginosa* strains PAO1 and PA14, while preserving cell viability, by antagonizing the QS receptors LasR and RhlR. The reduction of virulence factors and inhibition of biofilm formation have become recent strategies for preventing infections by multidrug-resistant bacteria because they do not interfere with cell viability, and hence, they put less pressure on bacteria to become drug-resistant through selection. Another study, by Passos et al. [21], evaluated the effectiveness of derivatives of the fruit and seeds of *Libidibia ferrea* for their antimicrobial and anti-adherence effects on *Streptococcus mutans* and for their inhibition of acidogenicity and the expression of GTF genes in *S. mutans* biofilms. Ethanolic extract, fractions, and compounds isolated from the plant material were studied. EG was among the isolated active compounds; it was shown to compromise biofilm formation by reducing the number of viable cells and by impeding the production of alkali-soluble glucans through the reduced expression of GTF genes. Compared to saline, EG reduced the expression of the *gtfB*, *gtfC*, and *gtfD* genes by 99.8%, 94.4%, and 99.7%, respectively. Acidogenicity and aciduricity were also reduced. 

We recommend the further testing of EG for its effects on other pathogens and on the expression of more genes known to be involved in biofilm production and maintenance. Additionally, the dual effect of EG on virulence factors and biofilm, as reported by Kim et al. [45], warrants further study. 

The results for the inhibition of biofilm formation by EG are summarized in Table 3.

## 5. Propyl Gallate

In their investigation of how several alkyl gallates affect virulence factors, biofilm formation, and QS in PAO1 and PA14 strains of *Pseudomonas aeruginosa*, Kim et al. [45] reported that propyl gallate (PG) exhibited more potent activity than ethyl gallate and butyl gallate in reducing biofilm formation and inhibiting the virulence factors elastase, pyocyanin, and rhamnolipid, without affecting the viability of the pathogen, by antagonizing the LasR and RhlR QS receptors. This indicates PG’s high potential for use against virulence factors and biofilm formation in *P. aeruginosa*. PG also countered the virulence of *P. aeruginosa* in the nematode *Caenorhabditis elegans* and a mouse model. All three gallates inhibited the production of QS signaling molecules and QS gene expression, with PG showing the strongest effects on both strains. PG was free of antibacterial effects in all the assays. Of the six alkyl gallates of interest in the study, only PG could be safely recommended to treat infections associated with biofilms in healthcare settings or to treat infections by drug-resistant *P. aeruginosa*.

Aracri, Cavalcanti, and Guimaraes [46] reported on a procedure using biofilm fermentation to produce tannase from biofilm formed by the food-contaminating mold *Aspergillus ochraceus*. By breaking down tannins, tannase can be used to assist in wine production, to improve the nutritional quality of animal feed, to stem pollution from the effluents in leather production, and to catalyze reactions in the synthesis of PG. The most fruitful method involved tannase production by *A. ochraceus* in Khanna medium, using gallic acid as the carbon source, followed by production using tannic acid, and the addition of yeast extract was shown to improve the yield of the enzyme. PG synthesized with tannase exhibited spectra similar to those exhibited by commercial PG.

Ding et al. [47] developed a virtual screening method for identifying substances that can be used to inhibit the QS regulatory system in bacteria that contaminate aquatic food products. Disrupting QS interferes with the cell’s gene regulation and intercellular communication and other collective behaviors, which can lead to more effective control of the pathogen and suppress food spoilage. A food-related, three-dimensional compound database was screened, and 25 promising substances, PG among them, were selected to be tested for their anti-QS properties against the bacterium *Pseudomonas fluorescens* P07. PG exhibited a markedly potent anti-QS action, which followed from its inhibitory effects on acyl-homoserine lactones (AHLs), signaling molecules used by QS bacteria to regulate a number of physiological functions.

Kosuru et al. [25] tested the capabilities of PG and GA to suppress pyomelanin synthesis and thereby affect biofilm formation in two clinical isolates of *Pseudomonas aeruginosa*. Pyomelanin plays essential roles not only in biofilm formation, but also in the virulence of the bacterium and in its resistance to oxidative damage, antibiotics, and its host’s immune response. PG was effective at preventing the growth of new biofilms and inhibiting the growth of pre-existing ones. However, with the administration of PG, and especially GA, pyomelanin secretion increased in the experimental strains, possibly due to the upregulation of synthesis in response to stress on the cell. No pyomelanin secretion was observed in non-adherent planktonic cells. The co-administration of ascorbic acid, however, led to reduced pyomelanin levels in the culture supernatants and synergized the inhibition of biofilm growth by GA and PG. 

The results for the inhibition of biofilm formation by PG are summarized in Table 4.

## 6. Butyl Gallate

Kim et al. [45], examining the effects of several alkyl gallates on virulence factors, biofilm formation, and QS in *Pseudomonas aeruginosa*, found that butyl gallate (BG), inhibited biofilm development and virulence factors (including elastase, pyocyanin, and rhamnolipid) in the *P. aeruginosa* strains PAO1 andPA14 by antagonizing LasR and RhlR receptors, although overall, the inhibitory effects were weak. Cell viability was not affected. BG also exhibited weak inhibition of QS gene expression and the production of QS signaling molecules.

## 7. Hexyl Gallate

In the study of BG previously mentioned, Kim et al. [45] also found that hexyl gallate differentially affected virulence factors. It weakly inhibited the production of rhamnolipid and pyocyanin, apparently by selectively inhibiting the RhlR system, weakly repressing the transcription of the *rhlI* and *rhlR* genes and genes involved in rhamnolipid and pyocyanin production. It had no effect on elastase production or biofilm formation. HG, as well as octyl gallate (OG), exhibited antibacterial activity, which prohibited the researchers from determining the effects of the two compounds on QS receptors.

## 8. Octyl Gallate

Oh et al. [48] observed synergistic anti-biofilm activity against methicillin-resistant *Staphylococcus aureus* (MRSA), using a combination of Octyl Gallate (OG), an antioxidant and food preservative taken from the medicinal plant *Terminalia bellerica*, and bacitracin, an antimicrobial peptide found in over-the-counter topical ointments. At a concentration as low as 10^−1^ U/mL, bacitracin alone significantly decreased MRSA biofilm development, but with the addition of 2 µg/mL of OG, significant reduction occurred at a concentration of 10^−3^ U/mL of bacitracin. The results demonstrate that at low concentrations, bacitracin, with OG as an adjuvant, exhibits marked MRSA anti-biofilm activity.

In a study by Gabe et al. [49], the effects of octyl gallate (C8-OG) were tested for the inhibition of biofilm formation by *S. mutans* on solid surfaces (polystyrene and glass) and for the inhibition of acidogenicity and the expression of genes essential to biofilm formation. The effects on biofilm development and acidogenicity were concentration-dependent. A concentration of 100.24 *µ*M completely suppressed biofilm growth on the solid surfaces and prevented 99% of the pH decrease seen in untreated bacteria. In addition, C8-OG significantly reduced the expression of four biofilm-related genes (*gbpB*, *gtfC*, *gtfD*, and *atpD*) and slightly decreased the expression of the *gtfB* gene. In planktonic cells, no significant impairment of gene expression was observed; however, there was a slight increase in the expression of the *atpD* gene.

In their investigation of how several alkyl gallates might affect virulence factors, biofilm formation, and QS in the PAO1 and PA14 strains of *Pseudomonas aeruginosa*, Kim et al. [45] observed, in their analysis of the production of QS signaling molecules and QS gene expression, that OG exerted differential effects on virulence factors. It reduced pyocyanin and rhamnolipid production by inhibiting the PqsR system but increased elastase production and biofilm formation by markedly stimulating the Las system. Antagonistic effects on QS receptors could not be determined due to OG’s antibacterial effect.

Saibabu et al. [50] investigated the antifungal effects of OG on *Candida albicans* and the mechanisms involved. They found it to be a strong inhibitor of the fungus. OG disrupts mitochondrial functioning in the cells, which triggers the production of reactive oxygen species (ROS); these cause lipid peroxidation and damage to cell membranes. OG was also found to compromise the metabolic flexibility of *C. albicans* and to inhibit virulence traits, including combating biofilm formation at all three stages of its growth and preventing the yeast-to-hyphae transition of the fungus. Nematodes (*Caenorhabditis. elegans*) infected with *C. albicans* and treated with OG showed improved survival rates. It was also demonstrated that OG showed potent antifungal activity not only against *C. albicans* but against other, non-*albicans* species of *Candida* as well.

Shi et al. [51] used OG in conjunction with photodynamic inactivation (PDI) by blue light (BL) to eradicate bacteria and eliminate biofilms of *Pseudomonas fluorescens*. In this study, PDI operated through the interaction of applied BL, the exogenous photosensitizer OG, and oxygen to produce ROS within the cell, which destroy intracellular lipids, proteins, and nucleic acids, and kill the bacterium. OG and BL administered individually exhibited some bactericidal effect, but BL irradiation with 0.4 mM of OG killed a significant amount of *P. fluorescens* cells in suspension, and *S. aureus* showed even higher susceptibility. BL also boosted the uptake of OG into cells, and BL + OG seriously damaged cell walls and caused cells to disgorge cytoplasm and collapse. Production of •OH resulted in lethal oxidative damage to lipids, proteins, and DNA. BL also enhanced the inhibition of biofilm formation by OG, and the combination was able to efficiently eradicate established biofilms. In a further experiment, the authors tested the efficacy of electrospun poly(lactic-acid) nanofiber-based packaging material in combination with OG and irradiated with BL for reducing the microbial contamination of giant salamander meat (a culinary delicacy and medicinal in traditional Chinese medicine) by *P. fluorescens*; 30 min of treatment reduced the bacteria count by approximately 99%, indicating that active OG/PLA nanofibers can be used to prolong the shelf life of perishable foods.

Shi et al. [52] investigated the bactericidal and antibiofilm effects of photodynamic inactivation (PDI) against the foodborne pathogen *Vibrio parahaemolyticus* using the combined action of OG, a food additive, and the application of BL. PDI was used on the pathogen, in both planktonic and biofilm growth forms, to target cell membranes and DNA. In the first stage, BL was used to promote OG uptake into the cells of *V. parahaemolyticus*, initiate the generation of toxic ROS, and cause the leakage of cell contents; in the second stage, BL irradiation, augmented by in situ OG, was used to extensively deconstruct cell membranes, proteins, and DNA, and the mechanisms underlying these processes were elucidated. It was shown that OG, as a PDI, is superior to conventional phenolic acids such as GA for inducing large amounts of protein degradation, in a process involving ROS and interference with the gene expression needed to synthesize essential enzymes, and killing bacterial cells. The combination of BL and OG also synergistically inhibited biofilm formation by *V. parahaemolyticus*. In an additional experiment, active packaging material incorporating nanofibers treated with OG was tested in combination with BL to assess its usefulness for preserving fresh salmon meat, demonstrating that this process can prevent contamination by foodborne pathogens over a number of days. 

The results for the inhibition of biofilm formation by OG are summarized in Table 5.

## 9. Dodecyl Gallate

Zhang et al. [53] tested the anti-microbial efficacy of five monomers found in traditional Chinese medicine, including dodecyl gallate (DG), against several pneumococcal strains of bacteria, among them *Staphylococcus aureus* and penicillin-resistant *Streptococcus pneumoniae* (PRSP). They targeted VicK/VicR, a two-component regulatory system in Gram-positive bacteria, which is essential to cell survival. All five compounds displayed antimicrobial effects against both penicillin-sensitive and penicillin-resistant strains of *S. pneumoniae*—in the latter case, especially DG and deoxyshikonin. Only these two agents inhibited the growth of *S. aureus* as well. The five compounds acted as strong bactericides on biofilm cells, with DG and deoxyshikonin inhibiting biofilm development at sub-MIC doses. Combined with penicillin, the two agents acted synergistically against all the drug-resistant strains of PRSP, both in vitro and in vivo, and showed synergistic antimicrobial activity against PRSP when used with erythromycin and tetracycline.

Gabe et al. [54] evaluated the effects of DG (lauryl gallate [C12-LG]) on five genes in *S. mutans* that are involved in biofilm formation (*gbpB*, *gtfB*, *gtfC*, and *atpD*), in relation to acidogenicity, gene expression, and biofilm development on solid surfaces (polystyrene and glass). In general, biofilm formation was significantly reduced by exposure to C12-LG (DG) in a dose-dependent manner compared to untreated controls. At a concentration of 98.98 *µ*M, biofilm formation was completely inhibited and pH levels were preserved. The suppression of acidogenicity was also dose-dependent. Four of the five genes tested showed no significant change; however, a significant effect was observed for *atpD*, in that exposure to a concentration of 77.1 µM of C12-LG produced a 48% decrease in fold change, and in planktonic cells, a 300% increase in fold change in the *atpD* gene was seen.

## 10. Conclusions

Several concluding remarks can be made in light of the results of the reviewed studies. The first is that alkyl gallates (gallic acid, methyl, ethyl, propyl, butyl, octyl, and dodecyl gallate) effectively prevent biofilm formation under doses below the MIC and are also capable of eradicating established biofilms. Second, the longer the alkyl chain length, the greater the antibiofilm activity of the gallic acid esters. Third, the mechanism of biofilm inhibition by the alkyl gallates likely involves suppression of the production of extracellular polymeric substances and QS signaling, as well as damage to the microbial cell membrane. Finally, the combination of alkyl gallates with other antimicrobial agents or physical factors, such as blue light irradiation, augments their antibiofilm activities in a synergistic manner, providing a rationale for using alkyl gallates in the treatment of infectious diseases, especially those caused by microorganisms resistant to antimicrobial drugs.

## Figures and Tables

**Table 1:** Gallic acid inhibition of biofilm formation by several bacterial species and its suggested mechanisms of action.

Bacterial Species	Effect of Biofilm Inhibition	Mechanism of Action	Dosage	Ref.	Note
*Proteus spp.* , *E. coli*, *Pseudomonas spp.* , *Salmonella spp.* , *Streptococcus mutans*, and *Staphylococcus aureus* and multispecies bacteria	Effective against planktonic bacterial growth and biofilm formation	Significant changes in extracellular polysaccharide	20–200 mg/L	[19]	No significant change on pre-formed biofilm
Clinical isolates of *Pseudomonas aeruginosa*	Effective in inhibiting the pre-existing biofilms	Possibly through the inhibition of pyomelanin synthesis	3–5 mg/mL	[25]	Synergistic effect of gallic acid and ascorbic acid seen in the inhibition of biofilm formation and associated pyomelanin synthesis
*Streptococcus mutans*	Effective against mature biofilm	Reduces the following: viable cells, production of alkali-soluble glucans, acidogenicity and aciduricity capacity, and expression of glycosyltransferase genes	at 250 µg/mL	[21]	
*Streptococcus mutans*	Effective against biofilm formation	Targeting the cell membrane		[27]	
*Chromobacterium violaceum*	Effective against biofilm formation	-	8.8 mM, 9.4 mM	[29,30]	Gallic acid does not affect QS
Clinical isolates of *Acinetobacter baumannii*	Effective against bacterial growth and biofilm formation	Cleavage of peptidoglycan, molecules-mediated quorum sensing, and the antioxidant activity of gallic acid probably implicated in regulating genes of biofilm formation	1.32–2.11 mg/mL	[31]	Significant association between MDR and the biofilm-forming ability of these isolates
*Ralstonia solanacearum*	Effective against young and mature biofilm formation	-	3 mg/mL	[32]	Gallic acid is an ecofriendly compound and could be used as a green pesticide
*Streptococcus mutans; Streptococcus oralis; Streptococcus mitis; Streptococcus salivarius*	Effective against bacterial growth	-	Less than 1.0 mg/mL	[34]	Gallic acid shows no cytotoxicity
Clinical isolates of *E. coli*	Gallic acid in combination with ampicillin is synergistically effective against bacterial growth and biofilm viability	Changes in membrane integrity and permeability of bacterial cell	The MIC value of gallic acid against *E. coli* (1024 µg/mL)	[38]	Gallic acid-ampicillin synergism
*Pseudomonas aeruginosa and Staphylococcus aureus*	Gallic alone and in combination with carvacrol inhibits mature biofilm	Changes in plasma membrane properties	MIC value 2.5 mg/mL	[39]	The effect observed with gallic acid and carvacrol combination seen also on dual-species mature biofilms of *S. aureus* and *P. aeruginosa*
Enteropathogenic *E. coli* [EPEC], enterohemorrhagic *E. coli* [EHEC], and enterotoxigenic *E. coli* [ETEC]	Effective against biofilm formation and its related genes	-	MBC value 2.1–2.2 mg/mL	[35]	
*E. coli*	Effective against planktonic bacterial growth and biofilm formation	Suppression of the pgaABCD genes	2 mg/mL	[36]	Inhibition of biofilm formation and biofilm eradication were at 2 and 8 mg/mL, respectively

(-) Mechanism of action was not reported in the reference.

**Table 2:** Methyl Gallate inhibition of biofilm formation tested on bacterial species, and its suggested mechanisms of action.

Bacterial Species	Effect of Biofilm Inhibition	Mechanism of Action	Dosage	Ref.	Note
*Streptococcus mu-tans*	Effective in preventing biofilm formation	By reducing the biofilm biomass, roughness and thickness	0.55–1 mg/L	[7]	Suppression of the biofilm acidogenicity
*Pseudomonas aeruginosa* (strain PAO1)	Effective in preventing biofilm formation	By inhibiting quorum-sensing gene expression and exopolysaccharide production	16–256 µg/mL	[40]	Decrease of the biofilm viability
*Streptococcus mutans*	Effective against biofilm formation	By inhibiting the bacterial adhesion to the dentin–resin interface	200–600 µg/mL	[41]	Epigallocatechin-3-O-(3-O-methyl)-gallate incorporated in the adhesive system
*Salmonella enterica Serovar* Typhimurium	Effective against bacterial growth and biofilm formation	By damaging bacterial cell membrane, reducing membrane potential and causing the leakage of bacterial cell contents	32–4096 µg/mL	[42]	The biofilm inhibitory effect of Methyl Gallate occurs in combination with antibiotic Tylosin, and the effect is synergistic
*Vibrio cholerae* (strains SG24 and PC4)	Effective against biofilm formation	By disintegrating the bacterial inner and outer membranes and leakage of cytoplasmic material	64 µg/mL	[43]	Methyl Gallate does not cause ≥90% reduction of biofilm at the 2 × MIC (128 µg/mL)
*Staphylococcus aureus*	Effective against biofilm formation	Possibly through the attenuation of bacterial growth	0.2 mg/mL, 0.4 mg/mL	[44]	Methyl Gallate does not potentiate the activity of antibiotic vancomycin against the biofilm-dwelling cells
Enteropathogenic *E. coli* [EPEC], enterohemorrhagic *E. coli* [EHEC], and enterotoxigenic *E. coli* [ETEC]	Effective against biofilm formation	By inhibiting expression of the biofilm-associated genes (*flhC*, *fliA*, *fliC*, *csgA*)	0.07–2.1 mg/mL	[35]	Low concentrations of Methyl Gallate inhibit the biofilm formation without significantly reducing cell populations

**Table 3:** Ethyl gallate inhibition of biofilm formation by several tested pathogens and suggested mechanisms of action.

Bacterial Species	Effect of Biofilm Inhibition	Mechanism of Action	Dosage	Ref.	Note
*Streptococcus mutans*	Effective against planktonic bacteria and in preventing biofilm formation	Significant changes in the gene expression of *gtfC* and *gtfB* and less on *gbpB*	2.78–3.53 mM	[6]	No significant effect on *gtfD*, *atpD*, and *atpF*
*Pseudomonas aeruginosa* (strains PAO1 and PA14)	Inhibited biofilm formation significantly	Inhibit virulence factor production and biofilm formation while preserving cell viability	3–30 μM	[45]	Among the key virulence reported factors: elastase, pyocyanin, and rhamnolipid
*Streptococcus mutans*	Inhibit biofilm formation	Significant reduction in the gene expression of *gtfB*, *gtfC*, and *gtfD*	50 mg/mL (252 mM) [EG was tested only against ATCC25175 biofilms]	[21]	Reduced the number of viable cells, acidogenicity, and aciduricity

**Table 4:** Propyl gallate inhibition of biofilm formation by several bacterial species and its suggested mechanisms of action.

Bacterial Species	Effect of Biofilm Inhibition	Mechanism of Action	Dosage	Ref.	Note
*Pseudomonas aeruginosa* (strains PAO1, PA14, and drug-resistant clinical isolates)	Effective against biofilm formation	By suppressing the production of extracellular polymeric substances, quorum-sensing signaling molecules, and quorum sensing gene expression	30–300 µM	[45]	Propyl Gallate inhibits the biofilm formation without affecting planktonic cell viability
*Pseudomonas fluorescens* (strain P07)	Possibly effective against biofilm formation	Possibly through inhibiting the production of quorum sensing signal molecules (acyl-homoserine lactones)	<2.25 mg/mL (anti-QS action in *Chromobacterium violaceum* CV026), <2.50 mg/mL (anti-QS action in *Agrobacterium tumefaciens* A136), not determined for *P. fluorescens* biofilm inhibition	[47]	Inhibition of quorum-sensing signal molecules occurs under sub-MIC
*Pseudomonas aeruginosa* clinical isolates (strains PA9027 and PA27853)	Effective in inhibiting the pre-existing biofilms	Possibly through the inhibition of pyomelanin synthesis	3–5 mg/mL	[25]	Synergistic effect of propyl gallate and ascorbic acid seen in the inhibition of biofilm formation and associated pyomelanin synthesis

**Table 5:** Octyl Gallate inhibition of biofilm formation by several bacterial and fungal species and its suggested mechanisms of action.

Bacterial and Fungal Species	Effect of Biofilm Inhibition	Mechanism of Action	Dosage	Ref.	Note
Methicillin-resistant *Staphylococcus aureus* (MRSA)	Effective against biofilm formation	By suppressing the production of extracellular polysaccharides	2 µg/mL	[48]	Synergistic effect of Octyl Gallate and bacitracin (10^−3^ U/mL) occurs in the inhibition of biofilm formation
*Streptococcus mu-tans*	Effective in preventing biofilm formation	By inhibiting the expression of biofilm-associated genes (*gbpB*, *gtfB*, *gtfC*, *gtfD*)	97.4–100.24 µM	[49]	Suppression of the biofilm acidogenicity through the inhibition of *atpD* gene expression
*Pseudomonas aeruginosa* (strains PAO1, PA14, and drug-resistant clinical isolates)	Enhancement of the biofilm formation	By increasing the production of extracellular polymeric substances	100–300 µM	[45]	Octyl Gallate reduces pyocyanin and rhamnolipid synthesis by inhibiting the PqsR system
*Candida albicans*	Effective in preventing biofilm formation and eliminating the preformed biofilm	By suppressing the transition of fungal cells from yeast to hyphae and damaging fungal cell membrane	20 µg/mL	[50]	Octyl Gallate causes mitochondrial dysfunction and induces oxidative stress in *C. albicans* cells
*Pseudomonas fluorescens*	Effective in preventing biofilm formation and eliminating the preformed biofilm	By inducing the production of reactive oxygen species and damaging the bacterial cell membrane	0.05 mM, 0.1 mM, 0.4 mM	[51]	Synergistic effect of Octyl Gallate and blue light (photodynamic inactivation system)
*Vibrio parahaemolyticus*	Effective in preventing biofilm formation and eliminating the preformed biofilm	By inducing the production of reactive oxygen species and damaging the bacterial cell membrane	0.1 mM, 0.2 mM	[52]	Synergistic effect of Octyl Gallate and blue light (photodynamic inactivation system)

## Sample Availability

Samples of the compounds are not available from the authors.
